# Infectious diseases specialists as clinical auditors: a lock-and-key fit

**DOI:** 10.1017/ash.2026.10860

**Published:** 2026-10-01

**Authors:** Angela Ishak, Nikolaos Mazonakis, Konstantinos Alexakis, Constantinos Tsioutis, Aris P. Agouridis, Nikolaos Spernovasilis

**Affiliations:** 1 Henry Ford Health System, USA; 2 Thoracic Diseases General Hospital “Sotiria”, Greece; 3 German Medical Institute, Cyprus; 4 European University Cyprus, School of Medicine, Cyprus

## Abstract

Clinical auditing is a structured quality improvement (QI) process that measures care against explicit standards, followed by implementation of change and re-audit. The effectiveness of the audit depends not only on the methodology but also on who conducts it. Infectious diseases (ID) specialists are equipped to excel as clinical auditors due to their combined expertise in clinical medicine, surveillance, infection prevention and control, antimicrobial stewardship, epidemiology, and cross-disciplinary leadership. Their integration with pharmacy, information technology, and facilities management departments equips them with a holistic skillset for hospital-wide QI. This review outlines the clinical auditing framework, contrasts it with research, QI and service evaluation, and presents a competency map for the ID specialists who act as clinical auditors. We propose high-yield audit domains where ID specialists’ leadership can drive measurable improvements in patient safety and organizational performance.

## Introduction

In modern times, a clinical audit can be defined as a quality improvement (QI) method that systematically reviews care against established standards and implements changes to improve outcomes.^1^ Unlike a research project, which aims to generate new knowledge and determine best practice, an audit seeks to find out if current clinical practice meets the defined standards and improves quality of care.^2^ It is characterized by a defined scope and a structured audit cycle, outlined in subsequent sections.

In complex hospital settings, effective auditors must be able to bridge clinical, administrative and technical domains. Infectious diseases (ID) specialists are well positioned to fulfill this role. Their responsibilities extend across most hospital departments, especially in the context of consultations requested by other specialties and due to their leading role in antimicrobial stewardship (AMS) and infection prevention and control (IPC) programs.^3,4^ This review outlines a structured framework for establishing the ID specialists as suitable clinical auditors within hospitals.

## The clinical auditor in modern healthcare: role and competencies

Clinical audit is a QI tool which aims to improve patient care and outcomes through systematically reviewing standard of care or current practices against explicit criteria, followed by measuring the outcome of the change with the goal of generating improvement.^5,6^ Although QI methods such as Plan–Do–Study–Act cycles emphasize rapid iterative testing, the audit cycle is anchored in re-measurement against explicit standards, ensuring that change is not only achieved but also sustained.^1,6,7^

The audit cycle consists of five core stages: (1) selecting a clinically relevant or safety-critical topic; (2) defining evidence-based standards; (3) measuring current practice; (4) comparing results to benchmarks and identifying gaps; and (5) implementing change followed by re-audit.^5,7^ Its structured nature allows organizations to demonstrate accountability and to close the loop between evidence and practice.

To achieve these aims, clinical auditors require more than technical measurement skills. Evidence appraisal and guideline translation are foundational, allowing international, national, or organizational standards to be converted into clear audit criteria.^5^ Additionally, metrics/healthcare literacy and risk adjustment ensure that indicators accurately reflect patient case mix and local context, while data governance and ethical awareness are instrumental to maintaining confidentiality and complying with regulatory frameworks such as the General Data Protection Regulation (GDPR) in Europe and the Health Insurance Portability and Accountability Act (HIPAA) in the United States.^7,8^ Equally relevant are interprofessional engagement and communication skills, as audits must be accepted by frontline staff to produce meaningful change.

Beyond these technical competencies, auditors rely on relational and implementation skills. Interprofessional communication, stakeholder engagement, and leadership are important for ensuring audit findings are translated into practice. A realist review of audit programs supported that success depends on local champions, bottom-up initiation, and clinical relevance, rather than externally imposed mandates.^9^ Similarly, practical frameworks highlight the value of stakeholder mapping, barrier anticipation, and resource planning to sustain improvement.^6^ Longitudinal evaluations confirm that auditors who embed re-measurement and structured feedback within institutional quality systems achieve the most durable improvements in care, underscoring the importance of sustained QI competency.^8^ Finally, at the highest level of audit maturity, the emphasis shifts from individual compliance to the analysis of unwarranted clinical variation, using measurement to reveal system-level inconsistencies that cannot be attributed to patient factors.^10^ This competency, interpreting data through a systems lens, allows auditors to translate metrics into organizational learning and sustained QI.

## Infectious diseases specialists as clinical auditors: evidence and rationale

**Figure 1.**
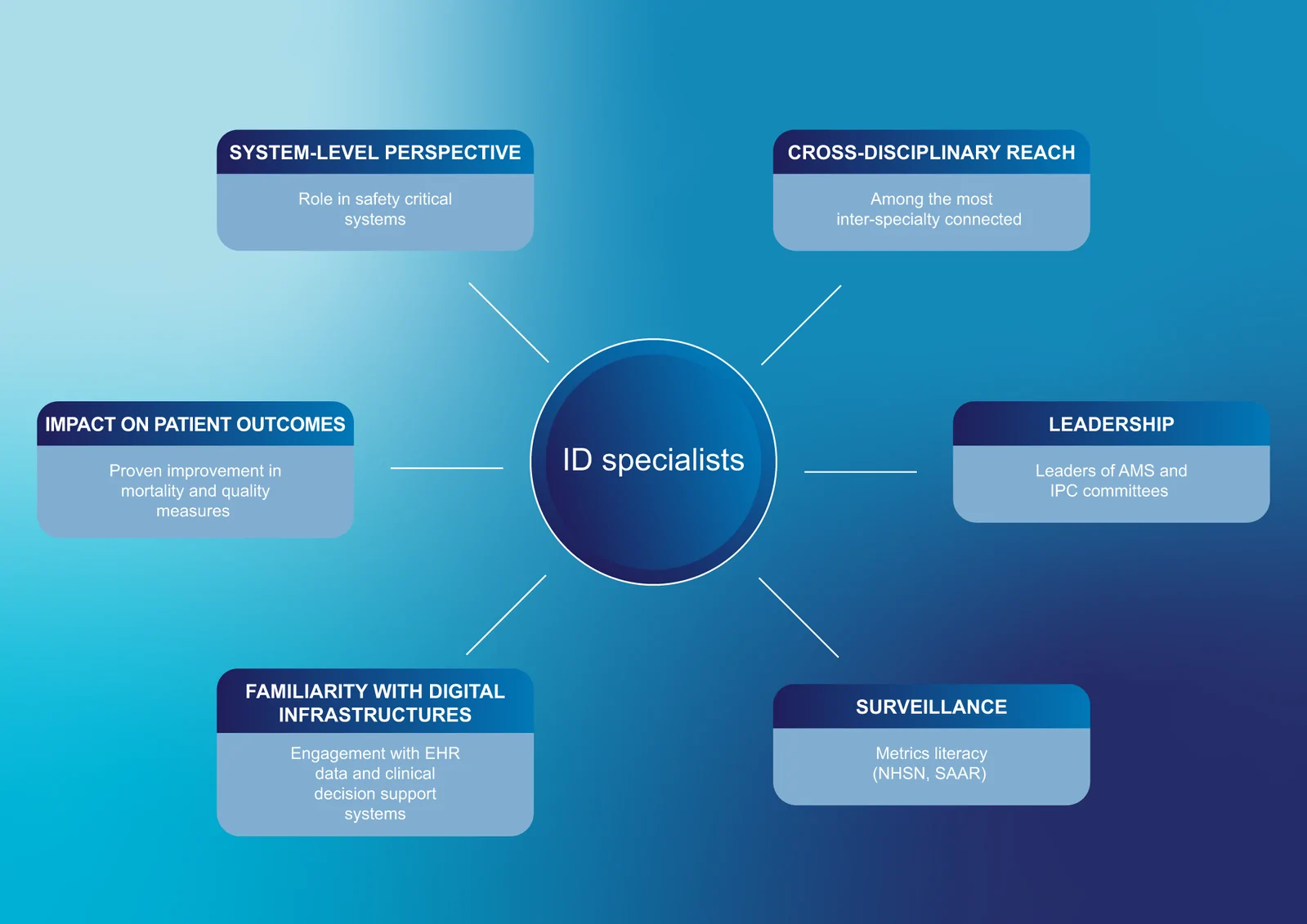
Distinctive qualities of infectious diseases specialists driving effective clinical audits.

ID specialists can contribute as clinical auditors due to their expertise in patient management, AMS, IPC, and healthcare systems. Their competencies in evidence-based medicine, measuring practice and multidisciplinary collaboration, provide a strong foundation. The competencies supporting this role are illustrated in Figure 1 and discussed in this section.

### Cross-disciplinary reach

A key characteristic of a clinical auditor is the capacity to engage in collaborative, interdisciplinary work with various clinical specialties and healthcare team members. Several studies have demonstrated that ID specialists’ consultations are requested by most specialties in a healthcare setting, including various internal medicine specialties, surgical specialties, as well as critical care medicine in the intensive care unit (ICU).^4,11,12^ Most ID specialists’ consultation requests are for diagnostic and therapeutic purposes in case of fever, elevated markers of inflammation without an apparent explanation, or an established infection, especially bacteraemia.^4,12^ Other common inquiries are related to the management of multidrug-resistant, difficult-to-treat, and opportunistic infections, as well as immunization and infection control issues.^12^ The necessity for ID specialists’ consultations becomes evident through studies that evaluate consultation patterns among medical subspecialties in a clinical setting.^13,14^ These include, among others, a study published by Jordan *et al*., where researchers examined administrative hospital discharge data for 3,979 patients who were hospitalized receiving care under the general medicine service over a 2-year period in two academic teaching hospitals in Canada, and a study that was carried out between June 2006 and December 2013 in a single academic hospital in Seoul, assessing the type of consultations requested by intensive care physicians in the medical, oncology, and surgical ICUs.^13,14^

The interdisciplinary influence of ID specialists in a hospital environment can also be explained by the complex nature of their patients, which requires competency, broad knowledge, resources, as well as collaboration with other specialties.^15^ In complexity analyses, patients seen by ID physicians ranked second overall, after nephrology, highlighting the level of interspecialty involvement required in their care.^15^ These findings underscore the need for interaction with other physicians in the management of complex patients and indicate that ID specialists are among the most interspecialty connected, supporting their role in system-level audits.

### Leadership in antimicrobial stewardship and infection prevention: a core competency for clinical auditing

Clinical auditors must possess leadership characteristics to ensure that audit findings translate into meaningful improvements. They need the ability to inspire engagement across diverse professional groups, foster a culture of accountability, and maintain credibility. Evidence indicates that ID physicians are able to assume such leadership mission, considering their role in the IPC and AMS programs.^16^ The World Health Organization (WHO) guidelines on core components of IPC Programs underscore the importance of ID physicians as infection preventionists.^17^ Furthermore, according to the “Core Elements of Hospital Antibiotic Stewardship Programs’’ updated by the Centers for Disease Control and Prevention (CDC) in 2019, the AMS program must have a leader or co-leaders including a physician and a pharmacist. This document highlights the effectiveness of a co-leadership model, as well as the value of ID expertise.^18^ Moreover, the Joint Commission has published a new AMS standard, emphasizing that AMS teams should include ID physicians, infection preventionists, pharmacists, and other practitioners, and highlighting that physician-led programs are successful.^19^ ID specialists are equipped with experience in the management of complex infections and proper use of antimicrobials, knowledge of traditional and new microbiological diagnostic methods, understanding of resistance patterns and public health needs, as well as the ability to influence prescribing habits of other physicians and drive QI, being appropriate for leadership of an AMS program.^20^

As leaders of these programs, ID specialists work in close collaboration with physicians of other specialties who regularly prescribe antimicrobials, nurses who inform program members about antibiotic administration and patients’ clinical course, and pharmacists who monitor antimicrobial consumption and play a major role in improving antimicrobial use.^18^ They are also in close contact with hospital epidemiologists and infection preventionists who provide surveillance data on multidrug-resistant and hospital-acquired infections (HAIs),^21^ microbiology laboratory staff who offer antimicrobial susceptibility reports and rapid molecular diagnostic methods contributing to diagnostic stewardship,^22^ information technology (IT) staff who incorporate program protocols and information regarding antibiotic use into electronic medical records,^23^ and hospital leadership and administration for organizational purposes and allocation of resources.^18^ Of note, many of these competencies are particular to ID physicians engaged in AMS and IPC programs and may not be uniformly represented across ID practice settings.

### Surveillance and metrics literacy

Surveillance is a cornerstone of ID practice and a natural extension of the clinical audit cycle. For instance, in the United States, ID specialists already oversee reporting to the National Healthcare Safety Network (NHSN), particularly through the Antimicrobial Use and Resistance (AUR) Module, which tracks antimicrobial consumption and resistance data.^24^ One of the most widely used benchmarking tools from this system is the Standardized Antimicrobial Administration Ratio (SAAR), a risk-adjusted metric comparing observed to predicted antibiotic use, considering patient case mix and facility characteristics.^25^ Because clinical audits require explicit, measurable standards, ID physicians’ familiarity with SAAR and other antimicrobial consumption metrics provides a validated framework for audit projects.^1,7^

Within these surveillance frameworks, high-yield audits that ID physicians can lead include assessments of empiric therapy appropriateness, time-to-appropriate therapy, IV-to-PO transitions, de-escalation practices, and treatment-duration concordance with established standards.^26,27^ These audits support compliance with organizational and regulatory expectations outlined in the CDC Core Elements of Hospital Antibiotic Stewardship Programs^18^ and allow benchmarking through systems such as the NHSN AUR Module^24^ and the European Surveillance of Antimicrobial Consumption Network (ESAC-Net).^28^

A comparable structure exists in Europe, where the European Centre for Disease Prevention and Control (ECDC) coordinates continentwide surveillance networks. The Healthcare-Associated Infections network (HAI-Net) monitors device- and procedure-associated infections, the European Antimicrobial Resistance Surveillance Network (EARS-Net) benchmarks resistance rates across member states, and the ESAC-Net tracks antimicrobial use using defined daily doses (DDD).^28,29^ As in the United States, these systems embed ID specialists in the processes of surveillance, benchmarking, and feedback.

In parallel, ID and IPC teams conduct and interpret surveillance of HAIs.^30^ These systems rely on standardized case definitions and denominators which are embedded in regulatory reporting systems worldwide.^17^ ID-led audit indicators of HAI prevention include insertion-bundle and maintenance-bundle adherence, documentation of device necessity, timely device removal, and device-utilization ratios. Because ID specialists routinely interpret risk-adjusted HAI metrics, they can translate audit findings into targeted interventions aligned with evidence-based recommendations.^30–32^ These audits also support compliance with organizational HAI prevention goals and accreditation standards.

Surveillance extends beyond microbiology; ID teams integrate pharmacy dispensing data, microbiology susceptibility patterns, and clinical documentation into composite metrics that guide AMS and IPC interventions. This ability to triangulate multiple data streams supports robust audits, which require validated data sources and consistent denominators.

### Digital and IT integration for high-leverage audits

As hospitals adopt electronic health records (EHRs) and increasingly embed clinical decision support systems, digital infrastructure has become central to the scalability and efficiency of clinical audit. ID specialists, already leaders in AMS and IPC and control activities, are able to leverage these tools to conduct valuable, data-driven audits.

Evidence demonstrates that computerized interventions can improve prescribing practices. A Cochrane review of inpatient AMS interventions confirmed that digital and decision support strategies improve antibiotic appropriateness and reduce unnecessary use.^33^ In addition, clinical decision support systems and real-time audit/feedback interventions have shown the greatest impact when seamlessly integrated into clinician workflows, ensuring higher uptake and sustained behavior change.^34,35^ More recently, digital stewardship tools have also been associated with reductions in broad-spectrum antimicrobial use and excessive treatment durations.^36,37^ These systems create standardized, reproducible data sets that fit seamlessly into the audit cycle. Drawing on their stewardship and IPC leadership, ID specialists are ideally placed to synthesize these metrics and convert them into targeted audits that inform both frontline practice and organizational policy.

Several randomized controlled trials further highlight the potential of digital integration for audits. The COMPASS trial, a cluster randomized control trial conducted in Swiss hospitals, showed that a multifaceted clinical decision support system combined with peer feedback improved the appropriateness of antibiotic prescribing, although effects on clinical outcomes were more modest.^38^ The WISCA tool (Weighted Incidence Syndromic Combination Antibiogram), which integrates local microbiology and resistance data into the EHR, tailored empiric therapy recommendations; in a crossover randomized control trial, WISCA was associated with improved prescribing appropriateness and reduced length of stay.^39^

Digital platforms also allow scalable auditing. Instead of manual chart review, automated EHR queries can identify prolonged antibiotic courses, perioperative prophylaxis given beyond 24 hours, or therapy discordant with culture results. These outputs can be directly integrated into dashboards and automated reports, providing clinicians with timely, individualized feedback, an approach consistent with Cochrane findings that feedback is most effective when salient, specific, and actionable.^34^

Nonetheless, challenges exist. Poorly designed alerts can contribute to alert fatigue, and some contextualized clinical decision support systems have shown limited effects on patient-level outcomes.^36^ Implementation also requires alignment with IT governance, adequate training, and integration into clinician workflows. Despite these barriers, ID physicians are able to lead digital audits, given their dual familiarity with prescribing practices and data-driven surveillance. By embedding audits into existing digital infrastructures, ID specialists can ensure audits are not only efficient but also sustainable and effective across the hospital ecosystem.

### Demonstrable impact of ID involvement on patient outcomes

The existing literature provides substantial evidence that ID consultation and interventions improve adherence to quality measures and patient outcomes.

#### Overall impact

Schmitt *et al.* evaluated the impact of ID specialists on outcomes of patients hospitalized from 2008 to 2009 in an acute care hospital with a diagnosis of 1 or more of 11 infections (bacteraemia, central line-associated bloodstream infections, *Clostridioides difficile* infection, endocarditis, vascular device infections, HIV/opportunistic infections, meningitis, osteomyelitis, prosthetic joint infections, septic arthritis, and septic shock), using Medicare claims data.^40^ They included 101,991 stays with and 170,336 stays without an ID intervention. After propensity score matching using regression models, ID versus non-ID intervention was associated with:

- significantly lower index stay mortality.
- reduced 30-day mortality.
- reduced 30-day re-admission rates.

Moreover, early ID intervention (within 2 d of admission) was associated with:

- significantly lower 30-day mortality.
- lower readmission rates.
- shorter hospital and ICU stays.
- lower Medicare charges and payments.^40^

In a subsequent retrospective analysis of privately insured patients younger than 65 years old who had an acute care stay in 2014 for selected infections, confirming 76,608 index stays and after cohorts were propensity score-matched for patient characteristics, patients who received early ID input were associated with:

- lower hospital mortality.
- shorter hospital stay.
- lower likelihood of readmission.
- lower total healthcare payments after discharge.^41^

#### Impact on Staphylococcus aureus bacteraemia (SAB) outcomes

ID consultation, especially if provided early during hospitalization, has been associated with:

- better adherence to evidence-based guidelines in the management of SAB, that is, appropriate antimicrobial therapy and treatment duration, early source control, obtaining of follow-up blood cultures and echocardiographic evaluation.^42–44^
- reduced short-term mortality.^42,45^
- improved long-term outcomes (ie reduced all-cause mortality and lower recurrence of infection at 5 years) in patients hospitalized with SAB.^46^

#### Other bloodstream and invasive infections

ID consultation has also been correlated with reduced mortality of patients hospitalized with enterococcal bacteraemia,^47–49^ gram-negative bacteraemia,^50,51^ and infections caused by drug-resistant *Enterobacteriaceae.* ^52^ Similar effects have been reported in patients with candidemia, as individuals receiving an ID evaluation have shown decreased mortality, primarily due to better implementation of evidence-based recommendations for the management of this complex infection.^53–55^

#### Impact on public health

The significant contribution of ID intervention to public health, implementation of best practices by healthcare professionals, decreased healthcare expenditure, and consequently, to improved patient care and outcomes has also been demonstrated by the effectiveness of AMS and IPC programs, in which ID physicians play a key role.^56,57^ The application of such programs has been associated to:

- reduced inappropriate antibiotic consumption.^58,59^
- decreased antimicrobial resistance.^59–61^
- lower incidence of *C. difficile* infection.^62^
- substantial healthcare cost-savings.^59,60^

These parameters are associated with enhanced quality of care and a lower incidence of complications due to multidrug-resistant pathogens during hospitalization, although effects on mortality are variable.^59,63,64^

#### Inpatient-outpatient interface: the case for outpatient antimicrobial therapy (OPAT)

Through OPAT, ID specialists play an important role in transition of care between inpatient and outpatient settings for patients with ID. Their expertise allows them to oversee the entire care pathway, reducing the risk of readmissions and enhancing the quality and safety of care outside the inpatient setting.^65^ OPAT is safe and effective, exhibiting mortality and treatment failure rates comparable to those of inpatient intravenous antimicrobial therapy, and ensuring significant healthcare cost-savings.^66,67^ As core members of OPAT programs, ID physicians can contribute to successful treatment of patients with ID, reducing the risk of HAIs, maintaining patient comfort and privacy, improving patient quality of life, and minimizing workload for healthcare personnel.^68^ Audits in OPAT also detect gaps in transitions of care, documentation, and outpatient–inpatient handoffs,^69^ enabling targeted improvements in safety and follow-up workflows.

Collectively, the aforementioned data suggest that ID-led audits can positively influence patient outcomes, mirroring the benefits achieved by ID specialists in the general clinical practice.

### Systems interfaces beyond clinical services

Many high-priority hospital audit domains extend beyond direct patient care and into the built environment, where the responsibility often rests with facilities, engineering, or environmental services. Despite this, their impact on HAI risk is substantial. ID specialists, by virtue of their leadership in IPC, can bridge these clinical–nonclinical interfaces and ensure that technical standards translate into measurable improvements in patient safety.

Water management is a prime example. Following recurrent outbreaks of *Legionella* and other waterborne pathogens, regulatory authorities such as the US Centers for Medicare and Medicaid Services (CMS) mandated that hospitals implement water management programs that include risk assessment, parameter monitoring, and documentation of corrective actions.^70^ Similarly, ASHRAE (American Society of Heating, Refrigerating and Air-Conditioning Engineers) Standard 188 provides detailed guidance on auditing hot-water temperatures, chlorine residuals, and flushing practices.^71^ In Europe, the ECDC has highlighted the importance of water safety plans and environmental monitoring within broader HAI prevention frameworks, supporting member states in implementing structured audits in line with national and WHO guidance.^72^ Although these activities are executed by facilities staff, ID specialists may provide the infection-risk perspective, determine thresholds for intervention and ensure findings are incorporated into hospital safety programs.

Environmental cleaning and disinfection represent another nonclinical but important audit domain. Fluorescent marker testing, ATP bioluminescence assays, and targeted cultures have been validated to measure cleaning thoroughness and correlate with reductions in *C. difficile* and multidrug-resistant organism transmission.^73,74^ ID specialists play a key role in validating audit tools, interpreting results in the context of HAI epidemiology, and linking them to clinical outcomes.

Air handling and ventilation standards also intersect with ID specialist audit leadership. Transplant and oncology units require high-efficiency particulate air filtration and positive-pressure environments, while tuberculosis wards or bronchoscopy suites rely on negative pressure. Audits of airflow rates and pressure differentials, though technical, directly affect risks of the transmission of multiple respiratory pathogens.^75^ ID specialists collaborate with engineering teams to define audit standards and translate compliance data into patient safety language for clinical and executive stakeholders.

**Figure 2.**
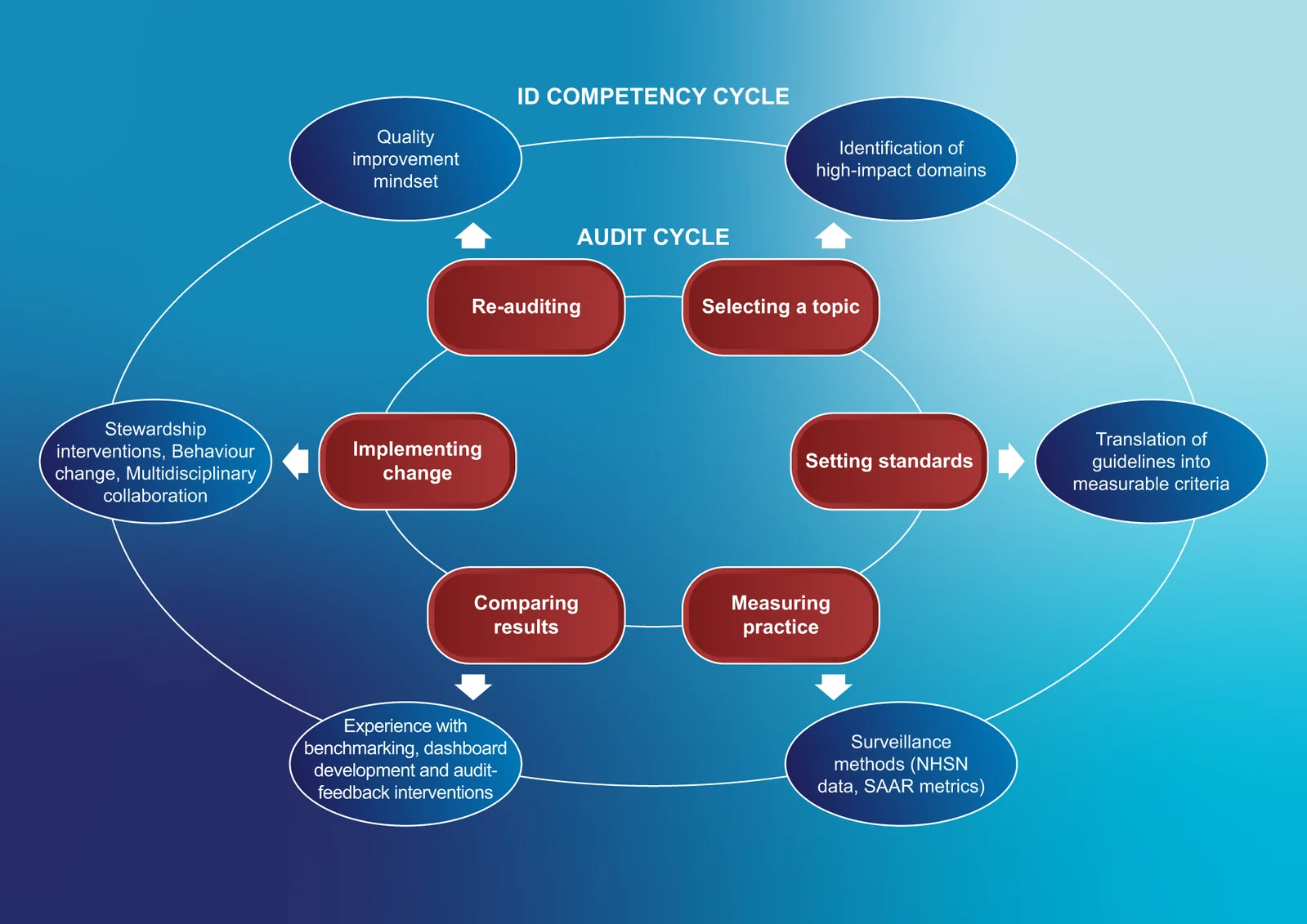
The ID competency cycle as a reflection of the audit cycle. Each step of the audit cycle can be effectively executed through the specific competencies of the ID physician.

In view of the foregoing, ID specialists are suitable to lead the clinical audit cycle. They are engaged in various domains such as antimicrobial resistance, infection control, or water safety, given their cross-disciplinary reach. Therefore, they are among the most suitable to select significant topics for clinical audit. In setting standards, ID physicians are adept in translating evidence-based guidelines into measurable criteria. This measuring practice aligns with their expertise in surveillance systems, metrics literacy, and use of standardized indices such as the NHSN and SAAR data. Comparing results is strengthened by their experience with benchmarking, dashboard development, and audit-feedback interventions. Their leadership in AMS and IPC committees, their ability to generate behavior change and their multidisciplinary collaboration with pharmacy, nursing, IT, and facilities management, make them appropriate to implement changes. Finally, re-auditing reflects their continuous QI mindset, supported by integration of digital health tools, updated guidelines, and cyclical stewardship programs. In this framework, an “ID competency cycle’’ is established, which effectively mirrors the audit cycle (Figure 2).

## ID specialists as clinical auditors: real-world experience

Real-world experience highlights the contribution of ID specialists as clinical auditors. The following examples are drawn from the audit activities of an Internal Medicine—ID specialist who also serves as the Clinical Lead Auditor in a 130-bed tertiary hospital, with a predominantly oncology case mix, leading a small team of well-trained in performing audits physicians, nurses, and supporting staff. The aforementioned team is responsible for the hospital’s AMS program through clinical rounds, surveillance of antimicrobial use and consumption, prospective audit and feedback, handshake consultations, and educational interventions, while also overseeing the IPC program including surveillance of HAIs, hand hygiene, and catheter care. In parallel, they conduct regularly several diagnostic stewardship audits that promote the rational use of diagnostic tests, including *C. difficile* testing as well as adjunctive biomarkers such as galactomannan, or molecular tests such as multiplex polymerase chain reaction diagnostics.

Several audit activities of this team extend beyond the ID sector. One example is an emergency oxygen administration prescription audit in hospitalized patients, in which the use of oxygen and compliance with prescribing guidelines is periodically assessed using a point prevalence survey methodology, already familiar from AMS and IPC audits. The findings of this audit led to review of existing policies, resulting in new protocols, staff education, and dentification of key gaps in practice requiring correction. Another example is the auditing of injectable medication procedures in all the wards of the hospital, through which gaps in nursing or medical practice have been addressed. This has also facilitated the recognition of IPC issues such as non-aseptic manipulation of central and peripheral catheters, highlighting the integration between general and IPC audits. Similarly, drug interaction audits in these wards have increased pharmacovigilance, while promoting AMS by identifying interactions between antimicrobials and other medications. Finally, under the leadership of the Internal Medicine—ID specialist, the aforementioned team of healthcare workers is also responsible for audits related to water and air quality, and environmental cleaning and disinfection.

## Conclusion

It is evident therefore that leadership and organizational skills, data integration and digital literacy, as well as transdisciplinary skills, communication of best practices and a high level of medical knowledge and current guidelines, are of paramount importance to translate into practice and QI measures across healthcare settings. The recognition of the ID specialists as persons of trust among both healthcare workers and hospital administration, the high degree of connectivity within the hospital, and the ability to integrate information from a wide array of medical and non-medical sciences into actionable plans make the ID specialists catalysts of what we already referred to as the “audit cycle.”
